# Protocol for the development of a multidisciplinary clinical practice guideline for the care of patients with chronic subdural haematoma

**DOI:** 10.12688/wellcomeopenres.18478.1

**Published:** 2023-09-01

**Authors:** Daniel J Stubbs, Benjamin M Davies, Mary Dixon-Woods, Thomas H Bashford, Philip Braude, Diedrik Bulters, Sophie Camp, Georgina Carr, Jonathan P Coles, Jugdeep Dhesi, Judith Dinsmore, Ellie Edlmann, Nicholas R Evans, Anthony Figaji, Emily Foster, Fiona Lecky, Angelos Kolias, Alexis Joannides, Iain Moppett, Mike Nathanson, Virginia Newcombe, Nicola Owen, Lisa Peterman, Amy Proffitt, Charlotte Skiterall, Peter Whitfield, Sally R Wilson, Ardalan Zolnourian, Meriem Amarouche, Akbar Ansari, Nick Borg, Paul M Brennan, Charlotte Brown, Christopher Corbett, Ruben Dammers, Tilak Das, Emily Feilding, Marilise Galea, Conor Gillespie, Laurence Glancz, Felix Gooding, Robert Grange, Natalie Gray, Peter Hartley, Taj Hassan, Dana Holl, Julia Jones, Richard Knight, Val Luoma, Harry Mee, Thais Minett, Stephen Novak, George Peck, Shvaita Ralhan, Jennifer Ramshaw, Davina Richardson, Ahmed-Ramadan Sadek, Katie Sheehan, Francoise Sheppard, David Shipway, Navneet Singh, Martin Smith, Rhonda Sturley, Michael Swart, William Thomas, James Uprichard, Vickie Yeardley, David K Menon, Peter J Hutchinson

**Affiliations:** 1 Division of Perioperative, Acute, and Critical care, University of Cambridge Addenbrooke's Hospital Cambridge, Cambridge, UK; 2 Healthcare Design Group, Department of Engineering, University of Cambridge, Cambridge, UK; 3 Department of Clinical Neurosurgery, University of Cambridge Addenbrooke's Hospital Cambridge, Cambridge, UK; 4 The Healthcare Improvement Studies (THIS) Institute, University of Cambridge, Cambridge, UK; 5 Department of Medicine for Older People, North Bristol NHS Trust, Bristol, UK; 6 Department of Neurosurgery, University Hospital Southampton, Southampton, UK; 7 Department of Neurosurgery, Imperial College Healthcare NHS Trust, London, UK; 8 Imperial College Healthcare NHS Trust, London, UK; 9 The Neurological Alliance, London, UK; 10 Department of Geriatric Medicine, Guy's and St Thomas' NHS Foundation Trust, London, UK; 11 Department of Anaesthesia, St George's University NHS Trust, London, UK; 12 Department of Neurosurgery, University Hospitals Plymouth NHS Trust, Plymouth, UK; 13 Department of Clinical Neurosciences, University of Cambridge, Cambridge, UK; 14 Department of Neurosurgery, University of Cape Town, Cape Town, South Africa; 15 Department of Clinical Neurosciences, Royal Infirmary of Edinburgh, Edinburgh, UK; 16 Department of Emergency Medicine, University of Sheffield, Sheffield, UK; 17 Department of Anaesthesia and Perioperative Medicine, University of Nottingham, Nottingham, UK; 18 Department of Anaesthesia, Nottingham University Hospitals NHS Trust, Nottingham, UK; 19 Department of Neurosurgery, Cambridge University Hospitals NHS Foundation Trust, Cambridge, UK; 20 ExEp Consulting, Calgary, Canada; 21 Department of Palliative Medicine, Barts and The London NHS Trust, London, UK; 22 Pharmacy Department, Manchester University NHS Foundation Trust, Manchester, UK; 23 Department of Anaesthesia and Critical Care, National Hospital for Neurology and Neurosurgery, London, UK; 24 Department of Neurosurgery, St George's Hospital, London, UK; 25 Department of Neurosurgery, University of Nebraska Medical Center, Omaha, Nebraska, USA; 26 Centre for Clinical Brain Sciences, University of Edinburgh, Edinburgh, UK; 27 Pharmacy Department, Cambridge University Hospitals NHS Foundation Trust, Cambridge, UK; 28 ACP in Emergency Medicine, Norfolk & Norwich University Hospital, Norwich, UK; 29 Neurosurgeon, Erasmus MC Stroke Center, Erasmus University Medical Center, Rotterdam, The Netherlands; 30 Consultant Neuroradiologist, Cambridge University Hospitals NHS Foundation Trust, Cambridge, UK; 31 Consultant Geriatrician (Major Trauma), Salford Royal Hospital, Salford, UK; 32 Department of Neurosurgery, Nottingham University Hospitals NHS Trust, Nottingham, UK; 33 Department of Emergency Medicine, St Thomas' Hospital, London, UK; 34 Department of Physiotherapy, Queen's Medical Centre, Nottingham University Hospitals NHS Trust, Nottingham, UK; 35 Department of Physiotherapy, Cambridge University Hospitals NHS Foundation Trust, Cambridge, UK; 36 Department of Emergency Medicine, Leeds Teaching Hospitals NHS Trust, Leeds, UK; 37 Department of Neurosurgery, Erasmus Medical Center, Rotterdam, The Netherlands; 38 The Burwell Surgery, Cambridge, UK; 39 Department of Rehabilitation Medicine, Cambridge University Hospitals NHS Foundation Trust, Cambridge, UK; 40 Department of Radiology, Cambridge University Hospitals NHS Foundation Trust, Cambridge, UK; 41 Department of Rehabilitation Medicine, North Bristol NHS Trust, Bristol, UK; 42 Department of Geriatric Medicine, Imperial College London, London, UK; 43 Department of Geriatric Medicine, Oxford University Hospitals NHS Foundation Trust, Oxford, UK; 44 Department of Neurosciences, Imperial College Healthcare NHS Trust, London, UK; 45 Department of Neurosurgery, Barking Havering Redbridge University Trust, Romford, UK; 46 Rehabilitation and Health Services Research, Kings College, London, UK; 47 Department of Emergency Medicine, Norfolk and Norwich University Hospitals NHS Foundation Trust, Norwich, UK; 48 Department of Emergency Medicine, Salford Royal NHS Foundation Trust, Salford, UK; 49 Department of Geriatric Medicine, St George's, University of London, London, UK; 50 Department of Anaesthesia, Torbay and South Devon NHS Foundation Trust, Torquay, UK; 51 Department of Haematology, Cambridge University Hospitals NHS Foundation Trust, Cambridge, UK; 52 Department of Haematology, St George's Hospital, London, UK; 53 Central London Community Healthcare NHS Trust, London, UK

**Keywords:** Chronic Subdural Haematoma, Neurosurgery, Perioperative Medicine, Protocol, Clinical Practice Guideline

## Abstract

**Introduction:** A common neurosurgical condition, chronic subdural haematoma (cSDH) typically affects older people with other underlying health conditions. The care of this potentially vulnerable cohort is often, however, fragmented and suboptimal. In other complex conditions, multidisciplinary guidelines have transformed patient experience and outcomes, but no such framework exists for cSDH. This paper outlines a protocol to develop the first comprehensive multidisciplinary guideline from diagnosis to long-term recovery with cSDH.

**Methods:** The project will be guided by a steering group of key stakeholders and professional organisations and will feature patient and public involvement. Multidisciplinary thematic working groups will examine key aspects of care to formulate appropriate, patient-centered research questions, targeted with evidence review using the GRADE framework. The working groups will then formulate draft clinical recommendations to be used in a modified Delphi process to build consensus on guideline contents.

**Conclusions:** We present a protocol for the development of a multidisciplinary guideline to inform the care of patients with a cSDH, developed by cross-disciplinary working groups and arrived at through a consensus-building process, including a modified online Delphi.

## Introduction

Chronic subdural haematoma (cSDH), a collection of aged blood within the subdural space, is especially common in older people living with frailty and other long-term conditions ^1– 3^. The exact aetiology is complex ^4^, since cSDH may arise with or without a history of antecedent trauma ^5^. People with asymptomatic cSDH may be managed conservatively, for example by stopping anticoagulation treatment and undertaking regular imaging, but the condition can enlarge to cause symptoms akin to a slowly progressive stroke. For those with symptomatic disease and acceptable surgical risk, surgical evacuation and subdural drainage can restore neurological function and may lead to normal survival relative to the general population ^6^. The incidence of operative cSDH is between 1.3–5.3/100,000/year, and when non-surgical cases are included the incidence of cSDH may be as high as 48/100,000/year ^6^. Incidence appears to be rising ^7^, with the demand for surgery projected to rise by at least 50% over the next 20–40 years ^6, 7^.

Organisation and delivery of cSDH care currently has no agreed best practice framework, introducing risks of unwarranted variation in practice and outcome. Care is delivered via a complex and often fragmented system spanning regional networks and professional and organisational boundaries, with patients receiving input from multiple disciplines across primary, community, secondary and tertiary care that may not always be well coordinated. Scheduling surgery (which needs to be undertaken in a tertiary centre) can be difficult, linked in part to pressured emergency pathways ^3^. Inpatient morbidity linked to cSDH is significant, though its true scope is yet to be characterised ^3, 8, 9^. Inter-hospital transfer for surgery (which needs to be provided in a tertiary centre) and for post-surgical rehabilitation is often necessary, further complicating pathways. In addition, outcomes for people who do not undergo surgery for cSDH are poorly understood ^10^.

Improving care for this common condition, which affects a vulnerable group, is a clear priority. Clinical practice guidelines, which provide statements of recommendations intended to optimise patient care, provide important frameworks for supporting best practice across both clinical and organisational aspects of care. Surgical care for older people in areas such as hip fracture has greatly benefited from evidence-based guidelines advocating multidisciplinary care, geriatrician-surgical co-management, and high quality perioperative care ^11, 12^. However, no such guidelines exist in the UK for managing cSDH along the clinical pathway from diagnosis, to surgery (or not), and back to the community.

Although there are similarities between such exemplar conditions and cSDH ^13^, a bespoke framework that addresses the tertiary nature of surgical care as well as the non-operative management of many people with cSDH is needed. Developing a cSDH-specific guideline, could, as well as defining current best practice based on the available evidence and the views of stakeholders, also identify knowledge gaps that could inform further research and help to inform targets for improving quality and safety.

In this protocol, we outline a study to develop a clinical practice guideline for cSDH led by the *Improving Care in Elderly Neurosurgery Initiative – ICENI* group. The programme began by seeking to understanding the needs and challenges of caring for people with cSDH ^14^, identifying areas of uncertainty as well as highlighting relevant learning from related surgical cohorts (such as hip fracture) ^14^ and identifying the diverse range of stakeholder groups that would need to be involved. This work determined that a co-design process should be used to develop a guideline for managing cSDH, with a particular focus on management for older people (those most likely to be affected by this condition).

ICENI is led by a Steering Committee, Management Group, and five thematic working groups. All members will complete a declaration-of-interests statement, covering both financial and intellectual aspects.

## Protocol

**Figure 1.**
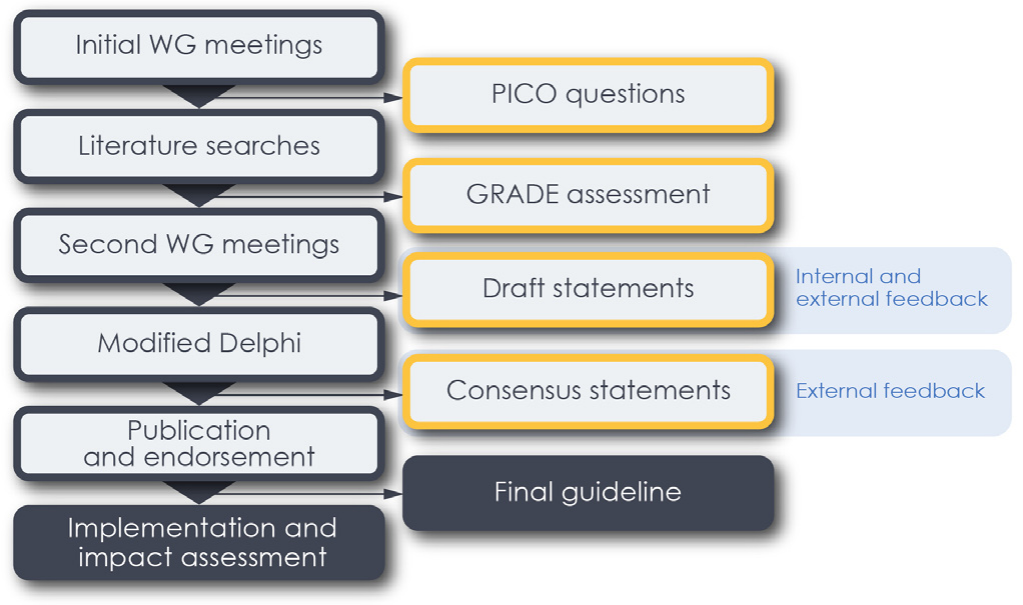
Flowchart of guideline development. Key methodological stages are shown in grey-bordered boxes, deliverables at each stage in orange-bordered. *WG = Working group*.

The guideline development process for this project ( Figure 1) aligns broadly with the processes used by the National Institute for Health and Social Care (NICE) ^15^ We have structured this methods section according to key stages of the Agree (Appraisal of Guidelines for Research and Evaluation) II Reporting Checklist ^16^.

### Scope and purpose

***Objectives*.** The proposed clinical practice guideline will seek to improve investigation, management and shared decision-making for adult patients (≥18 years of age) with suspected or definite cSDH by defining best practice based on current available evidence, specifying clinical pathways to enable better coordination of care, and supporting communication and shared decision-making with patients and carers.

***Questions*.** We seek to identify how care can be improved for people with suspected or confirmed cSDH at every stage along the pathway from the onset of symptoms, through diagnosis, treatment, perioperative care, and rehabilitation, as well as longer-term follow-up. This approach is consistent with other cross-disciplinary guidelines for the management of other surgical cohorts (such as hip fracture) ^11, 12^ but, for cSDH, with the added complexities of providing guidance to diverse specialties in different care settings.

***Population*.** The target population is people with cSDH. We define a cSDH as a pathological collection of aged blood and fluid within a subdural membrane ^4^. Ordinarily, cSDH is diagnosed using computerised tomography (CT) as mixed or hypodense crescenteric collections with or without a preceding history of trauma ^4^.

The guideline will consider stages relevant to the natural history of cSDH, including cSDH transformed from acute subdural haematoma (aSDH), iatrogenic CSDH and subdural hygroma (a collection of cerebrospinal fluid in the subdural space). The target population is characterised by older age ^6^, often with comorbidities (and/or frailty) but no group with cSDH will be excluded from the guideline.

### Stakeholder involvement

***Group and membership*.** The ICENI Steering Committee, formed in December 2021, has been constituted to oversee and coordinate the development of the guideline. It comprises a core programme group (including representatives from relevant national bodies and stakeholder groups) as well as the leaders of five thematic working groups. ( Table 1), Meetings will be held before and after each major project stage ( Figure 1) and at least every quarter. Meetings will be quorate if at least 50% of the committee, as well as 50% of stakeholder groups are able to attend. As appropriate, discussion may occur between meetings, for example, by email, with decisions approved at a steering committee meeting or by the chair as needed. A separate management group (BMD, DJS, EF, LP) will facilitate the day-to-day operations of this process and curate minutes from each working group meeting.

**Table 1.** Members of the ICENI steering committee and guideline management group, including their role and affiliations.

Participant	Affiliation	Role
Dr Thomas Bashford	Assistant Professor of Healthcare Systems, University of Cambridge Department of Engineering; Consultant in Neuroanaesthesia, Cambridge University Hospitals NHS Foundation Trust	Joint lead of Global Health working group
Dr Philip Braude	Consultant Geriatrician, North Bristol NHS Trust Foundation Trust Vice President of Age Anaesthesia Association	Joint lead of the Rehabilitation and recovery working group
Mr Diedrik Bulters	Consultant Neurosurgeon, University Hospitals Southampton NHS Foundation Trust	Joint lead of the non-operative and adjuvant management working group
Ms Sophie Camp	Consultant Neurosurgeon, Imperial College Healthcare NHS Trust	Joint lead of the rehabilitation and recovery working group
Ms Georgina Carr	President of the Neurological alliance	Steering committee representative (patient advocacy/charity)
Professor Jonathan Coles	Clinical Professor of Intensive Care Medicine, University of Cambridge Division of Anaesthesia, Consultant Neurointensivist, Cambridge University Hospitals NHS Foundation Trust, Research lead for the neuroanaesthesia and critical care society (NACCS)	Steering committee representative (anaesthesia/intensive care/research)
Mr Benjamin Davies	Doctoral research fellow Specialist registrar in Neurosurgery, Cambridge University Hospitals NHS Foundation Trust	Guideline coordination group
Dr Jugdeep Dhesi	Consultant Geriatrician, Guys and St Thomas’s NHS Foundation Trust Deputy Director of the Centre for Perioperative Care (CPOC)	Steering committee representative (medicine for the older patient)
Dr Judith Dinsmore	Consultant Neuroanaesthetist St George’s University Hospital, NHS Foundation Trust	Joint lead of the perioperative care working group
Professor Mary Dixon-Woods	Health Foundation Professor of Healthcare Improvement Studies, THIS Institute, Department of Public Health and Primary Care, University of Cambridge.	Steering committee representative (methodology and research)
Ms Ellie Edlmann	Academic Clinical Lecturer in Neurosurgery, University of Plymouth	Joint lead of the natural history and diagnosis working group
Dr Nicholas Evans	Honorary consultant in Stroke and Elderly medicine, University of Cambridge	Joint lead of the non-operative and adjuvant management working group
Professor Anthony Figali	Professor of Neurosurgery Immediate past president of the international neurotrauma society	Joint lead of the global health working group
Dr Emily Foster	Specialty doctor in Elderly medicine (neurosurgical liaison service)	Guideline coordination group Joint lead of the perioperative care working group
Professor Peter Hutchinson	Professor of Neurosurgery, University of Cambridge Research lead for the Royal College of Surgeons	Steering committee representative (Neurosurgery/research)
Professor Fiona Lecky	Professor of Emergency medicine Research lead for the Trauma and Audit Research Network	Steering committee representative (Emergency Medicine/TARN)
Mr Angelos Kolias	Honorary Consultant Neurosurgeon, University of Cambridge	Joint lead of the surgery and adjuvant therapy working group
Mr Alexis Joannides	Honorary Consultant Neurosurgeon, University of Cambridge	Lead of the implementation and audit working group
Professor David Menon	Professor of Anaesthesia, University of Cambridge Honorary Consultant Neurointensivist, Cambridge University Hospitals NHS Foundation Trust	Steering committee representative (anaesthesia/intensive care/research)
Professor Iain Moppett	Professor of Perioperative medicine, University of Nottingham Director of the Health Services Research Centre (HSRC)	Steering committee representative (perioperative medicine/research)
Dr Mike Nathanson	Consultant Neuroanaesthetist, Nottingham University Hospitals NHS Trust	Steering committee representative (Anaesthesia/Association of Anaesthetists)
Dr Virginia Newcombe	Honorary Consultant in Neurointensive care and Emergency medicine, University of Cambridge	Joint lead of the natural history and diagnosis working group
Ms Joanne Outtrim	Research Nurse, University Division of Anaesthesia, University of Cambridge BANN (British Association of Neuroscience Nurses) representative	Steering group representative: Nursing
Ms Nicola Owen	Neurosurgical specialist nurse, Cambridge University Hospitals NHS Foundation Trust	Joint lead of the rehabilitation and recovery working group
Dr Lisa Peterman	EXEP consulting	External facilitator and guideline coordination group
Dr Amy Proffitt	Consultant in Palliative Medicine President of the Association of Palliative Medicine	Steering group representative: Palliative medicine
Ms Charlotte Skiterall	Chief pharmacist, University Hospitals Manchester NHS Foundation Trust	Joint-lead of the perioperative care working group
Dr Daniel Stubbs	Clinical lecturer & Honorary Specialist Registrar in Anaesthesia, Cambridge University Hospitals NHS Foundation Trust	Guideline coordination group
Dr Sally Wilson	Consultant in Neuroanaesthesia and Neurocritical care University College London Hospitals NHS Foundation Trust President of the Neuroanaesthesia and Critical Care Society (NACCS)	Steering group representative (Anaesthesia/NACCS)
Professor Peter C Whitfield	Consultant Neurosurgeon, University Hospitals Plymouth NHS Trust President-elect of the Society for British Neurosurgeons (SBNS)	Steering group representative (Neurosurgery/SBNS)
Mr Ardalan Zoulnorian	Specialist Registrar in Neurosurgery, University Hospitals Southampton	Joint lead of the surgery and adjuvant therapy working group

Guideline development will be supported by The Healthcare Improvement Studies (THIS) Institute at the University of Cambridge ( https://www.thisinstitute.cam.ac.uk/), whose researchers will provide methodological guidance on co-design principles, guideline development and implementation, and by the Neurological Alliance ( https://www.neural.org.uk/),a charity established to represent patient needs in neurological healthcare policy.

Five thematic working groups ( Table 2) have been formed and structured around key themes, which were determined in a multi-stakeholder ICENI workshop held in October 2020. The groups will define the initial clinical questions to inform guideline development relevant to natural history and diagnosis, non-operative and adjuvant management, anaesthesia and perioperative optimisation, surgery, and rehabilitation and recovery. Further working groups will be established in the future to provide leadership for implementation and for global health application, particularly in low and middle-income countries, but these issues are not considered further in this protocol.

**Table 2.** Thematic working groups and proposed scope. CGA = Comprehensive Geriatric Assessment, cSDH = Chronic Subdural Haematoma, DGH = District General Hospital, ED = Emergency Department, MDT = Multidisciplinary Team, PACU = Postanaesthesia Care Unit.

Thematic working group	Focus	Stakeholders
Group 1: Natural History and Diagnosis LEADS: Ms Ellie Edlmann and Dr Virginia Newcombe	*Who gets a cSDH? Who will benefit from surgery? What do patients care about?* • Risk factors for cSDH and population epidemiology • Criteria for referral to neurosurgery and required information • Perioperative risk factors (e.g. using comprehensive geriatric assessment and shared decision-making • Patient/carer relevant outcomes • Patient and carer information requirements at different stages of care	**Primary Care** **Emergency Department** **Medical teams involved in diagnosis of cSDH** (e.g. acute medicine, stroke, geriatric medicine) **Ambulance service** **Patients/Carers** **Radiology**
Group 2: Non-operative and Adjuvant Management LEADS: Mr Diedrik Bulters and Dr Nicholas Evans	*What happens to those who don’t have surgery? What are the available non-operative treatment and management options and what is the evidence for them?* • Characteristics and outcomes of non-operative cohort • Role of conservative/adjuvant therapies and quality of evidence to support them • What are the criteria for reconsidering the need for surgical information? • Role of comprehensive geriatric assessment (CGA) in ongoing care • Care of patients in whom cSDH may represent a diagnosis at the end of life • Which patients subsequently require surgery? How are they identified and managed?	**Primary Care** **Medical teams** (e.g. acute medicine, stroke, geriatric medicine) **ED teams** **Neurosurgery** **Haematology** **Nursing/Care home providers** **Ambulance service** **Pharmacy** **Ward level care givers** (incl. postgraduate doctors, nurses, allied health professionals) **Non-medical teams involved in care of these patients** (e.g. trauma and orthopaedics) **Palliative Care** **Patients/Carers**
Group 3: Perioperative Care LEADS: Dr Judith Dinsmore, Dr Emily Foster, Ms Charlotte Skitterall	*How can pre-surgical care be optimised? What anaesthetic technique is best suited to cSDH? How can immediate perioperative complications be recognised and addressed?* • Transfer considerations, communication, safety • Recognition and treatment of acute medical conditions (e.g. delirium) prior to surgery and location of optimisation (DGH *v* tertiary centre) • Perioperative care pathway • Management of anticoagulation/antiplatelet agents • Optimal anaesthetic technique • Location and timing of optimization • Acute post-operative medical management • Escalation planning	Network coordination and transfer services **Medical teams** (e.g. acute medicine, stroke, geriatric medicine) **Haematology** **Neurosurgery** **Anaesthesia** **Critical Care** **Bed Managers** **Ward level care givers** (incl. postgraduate doctors, nurses, allied health professionals) **Commissioners** (re: Cost implications) **Patients/Carers**
Group 4: Surgery and adjuvant therapy LEADS: Mr Angelos Kolias, Mr Ardalan Zolnourian	*How should surgical technique and optimal post-operative care be defined?* • Surgical technique • Staffing • Postoperative disposition/PACU care • Post-surgical care (incl. anticoagulation management) • Scheduling • Post-operative imaging • Management of surgery specific complications (e.g. Recurrence, Infection, Pneumocephalus) • Decision making around the timing and composition of the team for surgery • Informed consent process • Adjuvant therapies (including middle meningeal artery embolization)	**Neurosurgery** **Anaesthesia** **Operating Department Practitioners/Theatre staff** **Ward level care givers** (incl. postgraduate doctors, nurses, allied health professionals) Critical Care **Interventional radiologists** **Patients/Carers**
Group 5: Rehabilitation and recovery LEADS: Ms Sophie Camp, Ms Nicola Owens, Dr Philip Braude	*What does ‘good’ recovery look like? How do we best perform rehabilitation?* • Physical and cognitive recovery • Role of wider MDT in specialist/non-specialist centres • Identification and management of those with ongoing rehab needs • Communication between healthcare providers • Appropriate discharge planning • Follow-up • Recognition of mid to late term complications (e.g. recurrence) and safety-netting • Consideration of burden of treatment for patients and carers and how it can be addressed	**Ward-level care providers** (e.g. postgraduate doctors, nurses, allied health professionals) **Medical teams** (e.g. geriatricians) **Psychology** **Pharmacy** **Discharge Planning/Bed managers** **Rehabilitation medicine** **Patients/carers** **Major trauma network/transfer representation** **Social workers** **Community services Primary Care**

Given the number of different organisations and professionals involved in care for cSDH, diverse stakeholder representation is important in guideline development. Therefore, to encourage breadth of perspective and depth of discussion, the thematic working groups will have a multidisciplinary composition ^17^ with two to three nominated leads, who will define the composition of their groups with reference to the specifics of their theme. Current stakeholders in the working groups ( Table 2) are listed by their major department and/or field, but each working group is expected to expand to include further representatives based on their setting of work, seniority (e.g., non-consultant *v* consultant grade), and medical, allied health professional, and nursing disciplines. Equality, diversity, and inclusion will be guiding values in recruiting to working groups.

Under the guidance of a professional and independent facilitator (LP), each working group will meet to identify key clinical questions within their theme of interest, including those framed using the *Population, Intervention, Comparator, Outcome (PICO),* structure. The groups will also define the scope of the required literature searches. Representative case studies will be developed to support and/or focus discussion. If, during discussion, provisional guideline recommendations as opposed to formulated clinical questions are generated, they will be subsequently converted into questions post-meeting by the guideline facilitation group.

Questions generated by each group will be circulated amongst the wider steering group for internal consultation. The views of patients and the public will be sought to ensure relevance, comprehensiveness, and attentiveness to the needs of patients and carers (see PPI section below for more information). Areas of overlap or underlap across the working groups will be coordinated at Steering Committee level to avoid duplication. All feedback will be reviewed by the steering committee and iterated as agreed. The final list of questions for evidence searching, and their scope, will be agreed by the steering committee.

Formal engagement of relevant professional bodies has also been identified as important in ensuring translation of guidelines into future clinical practice. Several have already confirmed their support for this endeavour and will be represented on the Steering Committee ( Table 3).

**Table 3.** Organisations engaged in guideline development with representation on steering committee.

Organisation	Stakeholder groups represented
British Association of Neuroscience Nursing (BANN)	- Nursing viewpoint (ward and critical care)
Neuroanaesthesia and Critical Care Society (NACCS)	- Neuroanaesthesia - Neurocritical care
The Neurological Alliance	- Patient advocacy
Society for British Neurological Surgeons (SBNS)	- Neurosurgery

***Target population preferences and views*.** Our project addresses themes identified as patient priorities in a 2015 priority-setting exercise conducted by the National Institute for Academic Anaesthesia (NIAA) and the James Lind Association ( https://www.niaa.org.uk/PSP). These include: ‘How can we improve recovery from surgery for elderly patients’ and ‘How can we improve communication between the teams looking after patients throughout their surgical journey?’

A specific patient, carer and lay panel will be convened to review research questions and draft recommendations made by the working groups and will have a key role in ensuring that at every stage the views of patients and carers are prioritised.

***Target users*.** The target users of our guideline are healthcare professionals involved in the care of patients with cSDH at any point throughout their journey from diagnosis to discharge. This includes clinicians from a variety of medical disciplines as well as allied health professionals involved in both inpatient and community care. Given the ‘hub-and-spoke’ model of care, our guideline will thus be of relevance to professionals across primary, secondary, and tertiary care.

### Rigour of guideline development

***Search methods*.** We will identify all primary literature and published systematic reviews pertaining to ‘Chronic Subdural Haematoma’, using index terms (e.g. MeSH), and both American and British English spellings. Searches will be conducted in Medline and EMBASE, supplemented by searches of the Cochrane database and performed following the advice of a medical librarian. Following input from our steering group we will include all studies since the inception of each database.

Initially all citations will be filtered for systematic review and/or meta-analyses, to identify existing reviews of relevance. To be considered relevant, a review must target the guideline question specifically, and obtain a high-quality rating on the AMSTAR2 tool ^18^. Identified systematic reviews were examined in a prospectively registered ‘Umbrella Review’ (PROSPERO ref: CRD42022328562) ^19^.

Results of searches will be de-duplicated and then titles and abstracts screened by at least two individuals before a decision on whether a study represents a primary observational or interventional study. Case-reports, narrative reviews, correspondence, and opinion pieces will be excluded. It is planned to exclude studies published in a foreign language. However, should a significant number of likely relevant articles be identified published in other languages, the feasibility of obtaining translations or summaries of the work may be explored at the discretion of the steering committee. Arbitration of any conflicts will be made by a third individual. Abstract screening will be performed using Rayyan.

***Evidence selection criteria*.** Review questions derived from working group discussions (in PICO format) will be grouped into relevant themes (e.g. those pertaining to surgical technique) and if no pre-existing systematic review of relevance is identified, registered on PROSPERO. These themes will be assigned to pairs of reviewers who will screen all primary studies to identify those of relevance. Conflicts will be resolved by a third reviewer where appropriate. In the first instance reviewers will identify interventional studies of relevance to the PICO question(s) relevant to their theme. Where no interventional or comparative studies of relevance are identified, the PICO question will be reformatted as a background level search for relevant observational evidence pertaining to the ‘intervention’ in question. For instance – if no comparative studies pertaining to the use of a post-surgical drain were identified, observational studies relating to drain use would be identified and their findings summarised for the screening group. All systematic reviews will be conducted in accordance with the PRISMA reporting guidelines ^20^.

Details on cohort characteristics will be extracted from identified studies but to be of relevance the study must report on the details of adult patients with a CSDH meeting our earlier definitions. In order to examine the relevance of available evidence to groups that may suffer healthcare inequality, data extraction will be performed with an awareness to key factors associated with differential health outcomes (PROGRESS-Plus) ^21, 22^.

We will include studies that include both patient level (e.g. recurrence, occurrence of medical complications) as well as system level outcomes (e.g. length of stay, time to surgery). Study quality will be assessed using a validated tool, selected based on the type of studies identified. Data extraction will use a piloted extraction template, of relevance to the study in question.

***Strengths and limitations of the evidence*.** Evidence will be synthesised into an evidence review for each question with data at the outcome level graded using the GRADE criteria. Bias in included studies will be assessed using the Newcastle Ottawa score (for retrospective studies) and the Cochrane risk of bias tool for randomised controlled trials (RCTs). Where possible evidence in the form of meta-analysis results will be included where studies of sufficient homogeneity and quality are found. Such analysis would be conducted as a random effects meta-analysis with heterogeneity tested using standard methods (I ^2^). If quantitative analysis is not possible then results will be synthesised in line with the SWIM recommendations ^23^

Where evidence for a particular question is not found, the steering group will consider the relevance of evidence from other conditions (including hip fracture ^11^ and safe transfer ^24^). Regardless of whether evidence from other conditions is felt to be of relevance, research questions with no primary evidence in cSDH will be considered in making final research recommendations in the final guideline.

### Formulation of recommendations (statements)

We will formulate recommendations in the form of statements.

***Draft recommendation production by thematic working groups and the steering committee*.** Each thematic working group will reconvene after literature searching is complete. In a second facilitated working group meeting, the output of each review question will be formulated into a draft clinical recommendation in the form of statements phrased according to recommendations from the National Institute for health and care excellence (NICE) ^15^.

**Figure 2.**
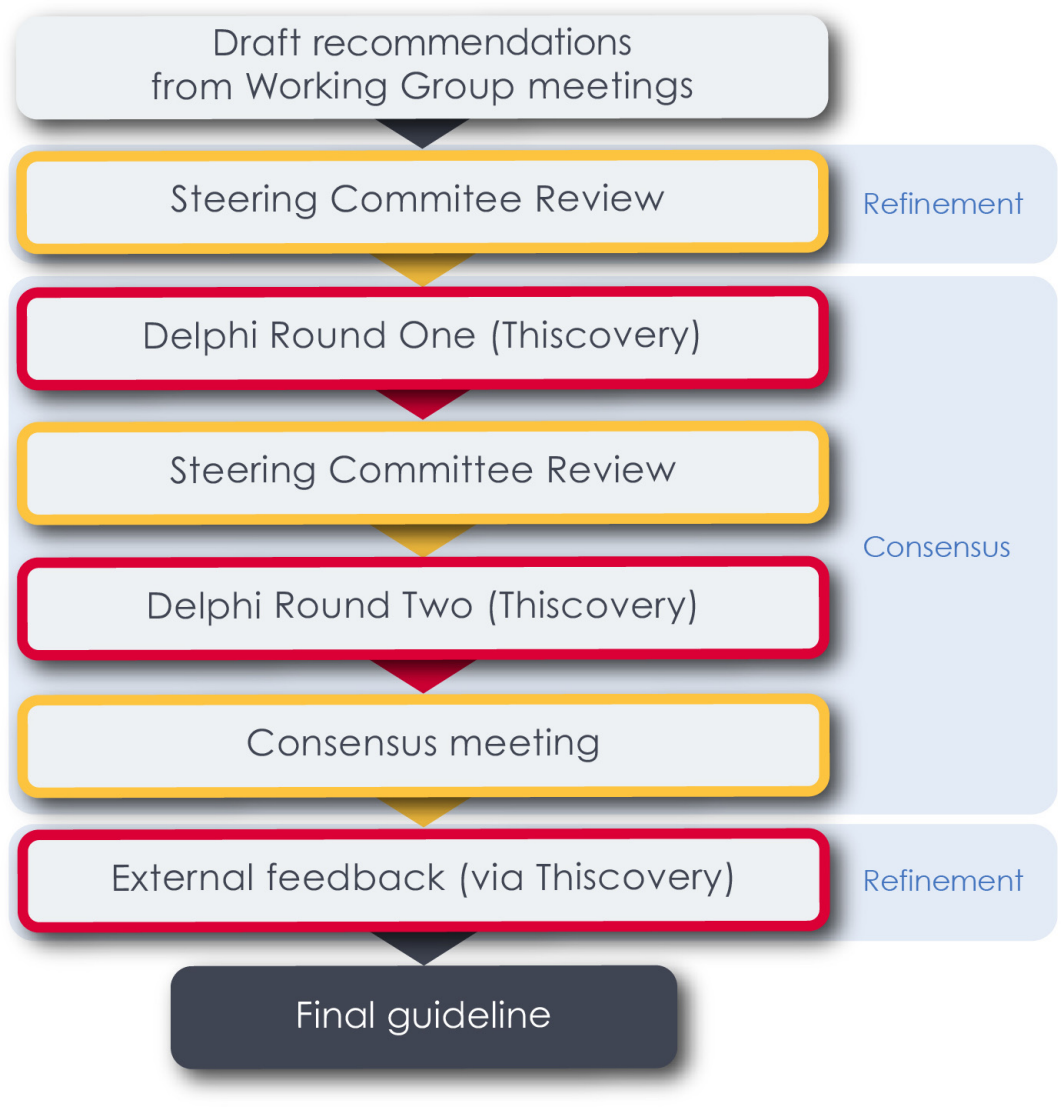
Translating draft recommendations from working groups into a final clinical practice guideline. Draft statements from working group meetings will be prioritised for inclusion in final guideline through an online Delphi consensus-building exercise, selection and finalisation by the ICENI steering group (yellow boxes), and a subsequent feedback and refinement on implementation and feasibility from external stakeholders. Red boxes indicate use of the Thiscovery platform.

Draft recommendations will then be subjected to a two-step process of refinement before being considered as part of a formal consensus process ( Figure 2). The first of these stages will be a review of each recommendation and its evidence by the steering committee, mainly focused on ensuring that each recommendation is within the scope of the process and is clearly phrased. The latter phase will seek external feedback for which appropriate approvals will be sought.

***Consensus-building Delphi exercise on statements to be included in the clinical guideline*.** We will use a modified Delphi involving a broad range of stakeholders ( Table 3) ^23^ to prioritise clinical practice statements for inclusion in the guideline through two rounds of surveys that will iteratively build consensus.

Consensus-building methods such as Delphi are well established as ways of promoting deliberation, inclusion, and participation in situations where there may be multiple perspectives, interests and communities. Though Delphi exercises have potential to include a large number of individuals across diverse locations and areas of expertise, many exercises for healthcare have involved relatively small and homogeneous panels of approximately 10 to 30 participants. There is evident scope for including larger and diverse groups of participants, particularly through use of online methods, which we will seek to utilise.

Our Delphi ^25^ will be hosted on Thiscovery, which has already supported similar exercises ^26^. An online platform developed by THIS Institute for supporting research, development, engagement and consultation in healthcare, Thiscovery is founded in an ethos of co-creation and participation. It provides an inclusive and rewarding way of engaging the knowledge, skills, creativity, lived experience and expertise of multiple stakeholders, including patients, carers, NHS staff, experts and many others. Thiscovery can be accessed on PCs, phones, tablets and other devices, and been designed to comply with level AA Web Content Accessibility Guidelines, and uses bespoke images specifically designed to represent diversity. The outcomes of the Delphi exercise will be reviewed and ratified at an in-person meeting of the steering committee ( Figure 2).

Participants in the Delphi will be healthcare professionals. The option of including patients and carers in the exercise was given extensive consideration, and it was concluded that the length and technical nature of the questionnaires rendered them inappropriate. Instead, patient and carer feedback on the draft statements will be provided by patient and public involvement (PPI) representatives throughout the process, including the final in-person meeting to review and ratify the statements.

To be eligible to participate in the Delphi ( Table 4) participants must be aged ≥18 years old, be able to give informed consent and be a healthcare professional involved in the care of cSDH ( Table 5). Rationale for inclusion of specific groups is given in Table 5. Delphi participants for Round 1 will be recruited using the networks of the steering and working groups, professional societies (e.g. the neuroanaesthesia and critical care society (NACCS)) and, where appropriate, social media.

**Table 4.** Eligibility criteria to participate in a Delphi to provide feedback on draft clinical care statements for a chronic subdural haematoma (cSDH) guideline.

** Eligibility criteria for Delphi** 1. Participants whose role is primarily is to provide care for people with a suspected or confirmed cSDH (Eligible for rounds 1-3) a. Age: ≥18years b. Consent to participate c. Primary role: healthcare professional **Eligibility criteria for ‘Feasibility of implementation’ survey** 2. As above **and** 3. Participants whose primarily role is as a system stakeholder involved in making decisions or about organisation and delivery of care. (Eligible for round 3) a. Age: 18+ years b. Consent to participate c. Primary role: Decision-maker at ICS, regional or national level 4. Participants who are healthcare stakeholders involved in advocating for the population affected by cSDH (Eligible for round 3) a. Age: 18+ years b. Consent to participate c. Do not directly provide patient care but work in national bodies, audits, charities and other groups with a role in relation to the population affected by cSDH

**Table 5.** Stakeholder groups from which views will be sought on proposed recommendations to be included in the guideline. MDT = Multidisciplinary team.

Stakeholder Group (* indicates will aim to capture perspectives from secondary and tertiary care)	Rationale
Anaesthesia and Critical Care	Delivery of anaesthesia to this patient group
Commissioners/managers*#	Funding of new services
Emergency Medicine and Acute Medicine*	Involved in initial diagnosis/referral to neurosurgery
Dieticians	MDT management of older surgical patients
Haematology*	Management of perioperative anticoagulants/antiplatelets
Geriatric medicine*	Initial diagnosis, management post repatriation, potential for surgical liaison role
Neurosurgery	Delivery of the care pathway and surgery
Nursing*	Ongoing patient care, communication, family liaison.
Occupational Therapy*	Assessment of function and cognition, maximising postoperative functional recovery
Palliative Care*	For those patients whose diagnosis may mark a transition to a best supportive or symptom focused treatment approach.
Pharmacy*	Medicines reconciliation/optimisation including safety on inter-hospital transfer
Physiotherapy*	Assessment of function, balance, mobility, and recovery postoperatively. Avoidance of deconditioning
Rehabilitation Medicine*	Rehabilitation strategies and outcomes
Speech and Language Therapy	MDT management of older surgical patients
General Practice	Community care, follow-up, diagnosis.

Individuals will be asked to indicate the importance and/or suitability of each recommendation for inclusion in the guideline. It will be made clear that statements should be voted on purely in terms of whether they reflect the participant’s view of clinical best practice (and not, for example, feasibility). Individuals can indicate whether a statement should be: *included as written*, *included with amendment*, or *excluded*. Free-text comments will be sought for responses that are not “include as written”.

 A statement will be considered for inclusion in the guideline if more 66% respondents to that question indicate a statement should be included in its current form. Exclusion will be more stringent, with 75% of respondents having to indicate a statement should be excluded. If a particular question is answered by 20%% or fewer of total participants the inclusion decision for this will be highlighted to the steering committee for consideration of discussion.

Analysis of free-text suggestions will be based on principles of content analysis ^27^ ( Table 6), with the initial coding framework including the following categories:.

**Table 6.** Approach to handling free-text suggestions.

Type of suggestions	Action
**Typographical changes**	Comments relating to minor issues of grammar, punctuation and spelling will be noted and where necessary amendments will be made. For purposes of determining consensus votes exclusively focused on making such suggestions will be counted in the ‘ *include as is’* category.
**Changes to the strength of recommendation**	Suggestions relating to the strength of the statement (e.g. *should* rather than *could*) will be identified and the statement considered for rephrasing. Rephrasing will occur if the number of suggestions for the new wording exceeds the number of votes for including the statement ‘as is’, or if there appears to the Steering Committee to be a good reason to make the rephrasing.
**Content suggestions**	Changes that appear to propose changes to the content of a statement (e.g. change to a suggested time frame or inclusion or deletion of a component of a statement) will be handled as changes in statement strength above.
**Other statements (e.g. those in support of exclusion)**.	Any other changes will be noted and presented to the steering group to support their decision making (either between rounds 1 and 2 or at the consensus meeting).

Findings of the first round will be reviewed by the Steering Committee to confirm which statements will be carried forward to Round 2 and how statements should be rephrased (if applicable). To support decisions on rephrasing, a summary of the coding of free-text suggestions will be presented to the Steering Committee. A simple majority of response will be used where needed if consensus cannot be reached through discussion.

In the second round, participants will be presented with statements on which there was not yet consensus for inclusion (out of those they addressed in round one), together with narrative reporting of free-text suggestions from the first round if applicable. Responses for indicating inclusion will be the same as in Round 1. The same thresholds will be used to determine whether statements have met the criteria for inclusion or exclusion. Statements will be considered for rephrasing in advance of the in-person meeting using the same criteria as in round 1.

***Consensus building: in-person meeting*.** Following Round 2, we will convene a consensus meeting of the steering group and relevant disciplinary representatives from working groups and patient, public and carer representation to ensure all appropriate viewpoints are represented. All statements proposed for exclusion and inclusion in the guideline as an outcome of the Delphi process will be reviewed by members in advance of the meeting. Any item can be suggested by attendees for formal discussion at the in-person meeting. The meeting will seek to obtain consensus on ‘edge cases’ where formal consensus has not been reached in the preceding two rounds of questionnaires. Any statement selected for discussion will proceed to inclusion in the final guideline following a simple majority decision in situations where it is not possible to achieve consensus through discussion.

***Survey of influences on implementation*.** Preparing for implementation is a key stage of guideline generation. We will establish a specialist working group to identify how implementation of the guideline can be optimised and to inform development of an ongoing audit infrastructure. The implementation working group will draw upon experience from similar initiatives in other clinical settings, including ongoing national neurosurgical audit projects ( https://www.sbns.org.uk/) and include experts in implementation science ( Figure 2).

A survey will be designed to access views on implementation strategies for the clinical statements proposed for inclusion in the clinical guideline. The survey will be distributed to those who completed the earlier Delphi survey, as well as steering and working group members. We will also seek to recruit key stakeholder groups ( Table 5) including, for this survey, those in managerial roles and who work for patient-supporting organisations and charities.

Participants will be shown each clinical statement and asked to rank its ‘ease of implementation into practice’ on a five-point Likert scale. Free-text comments on what might influence implementability will be sought. Findings from this survey will be presented to the working group on implementation and to professional bodies (e.g. the society for British neurological surgeons - SBNS) who will review the guideline for subsequent endorsement.

***Consideration of benefits and harms*.** Following evidence synthesis and before the commencement of the Delphi, the steering committee will consider the scope of purported health benefits of specific interventions. They will also review the potential risks of discrimination or exclusion of vulnerable groups if specific recommendations were to be enacted. Decision-making will be informed by the results of the critique of available evidence using the PROGRESS-PLUS criteria. Recommendations felt to be at risk of inducing future inequities or other unintended consequences will either be excluded or will be enhanced with alerts about the potential risks and will be highlighted as a priority for future research to ascertain the potential for inequity and strategies for its mitigation.

***Link between recommendations and evidence*.** Each recommendation will be linked to relevant PICO questions, supporting evidence strength (using GRADE), and links to relevant publications. This information will be included in an appendix to the final guideline to ensure appropriate transparency and will be made available to Delphi participants.

***External review*.** Our guideline will be developed with appropriate external contributions from a broad range of relevant stakeholders ( Table 3), supported for some elements by the use of a novel consultative platform (Thiscovery). This will provide valuable critique at two junctures: first, in the prioritisation of draft statements for inclusion in the final guideline, and second, by providing additional review following our final consensus meeting to give an initial view on implementation strategies for the guideline. Feedback will be collated both through structured use of a Likert scale as well as free-text comments.

***Specific and unambiguous recommendations*.** Recommendations formed by the working groups will aim to be action-orientated and to suggest measurable activities. Statements will seek to describe when, by who and to whom, what should occur, with a level of obligation (e.g. must, should or may) ^15^. They will explicitly include consideration of benefits and harms, including any risks to equity and inclusion, and the profile of trade-offs of benefits and harms. Each recommendation will include a clear description as to which patient group it applies (e.g. patients awaiting surgery, patients triaged to non-operative care).

***Management options*.** Recommendations will advise on management decisions (including surgical technique and alternative strategies) for patients with cSDH throughout their journey from diagnosis to ultimate discharge.

***Identifiable key recommendations*.** Presentation of the final guideline will ensure that key or safety-critical recommendations are appropriately highlighted with recommendations pertaining to specific staff groups (e.g. neurosurgeons, nursing staff, geriatricians, and allied health professionals) grouped together for ease of reference.

### Applicability

***Applicability to low- and middle-income countries*.** Though the initial guideline is intended to be primarily applicable to the UK (and thus potentially other high-income countries), the distinctive needs and features of low-and-middle-income (LMIC) countries were recognised and will be addressed in subsequent work. We have already appointed specific steering group representatives who will lead this stream of work at the completion of the UK workstream described in this paper.

***Resource implications*.** As part of future work, we will conduct an appropriate health economic assessment that is cognisant of the distributed nature of cSDH care across institutions.

***Monitoring/auditing criteria*.** We will ensure the development of all necessary implementation tools as recognised by NICE ^17^.

### Editorial independence

***Funding body*.** This project is funded by the Association of Anaesthetists/Anaesthesia via the National Institute for Academic Anaesthesia (NIAA) (WKR0-2021-0014) and the Health Foundation’s grant to The Healthcare Improvement Studies Institute (THIS Institute), with early work funded by the Addenbrookes Charitable Trust (ACT) (900268). Details of individual awards held by senior and corresponding authors are given in ‘Grant information’.

***Competing interests*.** All members will declare conflicts of interests, covering both financial and intellectual aspects in all publications.

***Updates*.** A guideline update will be considered five years following publication but might be considered sooner should need be identified, for example as new evidence or techniques become available.

### Patient and public involvement

Appropriate patient and public involvement on our working groups has been obtained with additional oversight from a patient facing charity (The Neurological Alliance).

### Ethics and dissemination

The ethical risks of this project are low, since it is primarily consultative and does not include intervention. The participants in the consensus-building exercise are healthcare professionals who are unlikely to be exposed to risk of harm through completing the questionnaires. Individuals will be asked to indicate their consent to participate in this consultation via the online platform.

Patients and carers will be integral to the output of our working groups and are integrated into the research team ^28^ but, because of the technical nature of the clinical guideline statements, will not be involved in the Delphi consensus-building exercise.

Dissemination of our guidelines is of paramount importance. Final guidelines and literature reviews will be published open access in scientific journals as well as being hosted in institutional repositories where appropriate. It is envisioned that dissemination via annual scientific meetings across relevant disciplines will also occur.

Ethical review was undertaken by the University of Cambridge psychology ethics committee (PRE.2023.065). Favourable opinion was received on the 26 ^th^ May 2023.

### Study status

At the time of protocol publication in August 2023 our consensus building Delphi is in progress.

## Data Availability

No data are associated with this article. Open Science Framework: PRISMA-P checklist for ‘Protocol for the development of a multidisciplinary clinical practice guideline for the care of patients with chronic subdural haematoma’. https://osf.io/phy3k/ ^20^ Data are available under the terms of the Creative Commons Zero “No rights reserved” data waiver (CC0 1.0 Public domain dedication).
