# Supplementary material for: Protocol for the development of a multidisciplinary clinical practice guideline for the care of patients with chronic subdural haematoma
Source: Wellcome Open Res. 2023 Sep 1;8:390. [Version 1] doi: 10.12688/wellcomeopenres.18478.1 (PMC10905132; doi:10.12688/wellcomeopenres.18478.1)
Supplement: Peer review history

# Reviewer report: Reviewer response for version 1

**DOI:** 10.21956/wellcomeopenres.20492.r72473

**Published:** 2024-02-13

**Authors:** Sae-Yeon Won

**Affiliations:** 1 Department of Neurosurgery, University of Rostock, Rostock, Germany

All in all, it is a very important step to develop a clinical practice guideline for the care of patients with cSDH. We all face different management and expert opinions across the clinics and national boundaries, and this will help to uniform the pre/intra- and postoperative management of this frequent disease.

Is the study design appropriate for the research question?

Yes

Is the rationale for, and objectives of, the study clearly described?

Yes

Are sufficient details of the methods provided to allow replication by others?

Yes

Are the datasets clearly presented in a useable and accessible format?

Yes

Reviewer Expertise:

subdural hematoma, traumatic brain injury, vascular neurosurgery, stroke

I confirm that I have read this submission and believe that I have an appropriate level of expertise to confirm that it is of an acceptable scientific standard.

# Reviewer report: Reviewer response for version 1

**DOI:** 10.21956/wellcomeopenres.20492.r72462

**Published:** 2024-02-13

**Authors:** Atakan Orscelik

**Affiliations:** 1 Department of Neurological Surgery, University of California San Francisco, San Francisco, California, USA

The article aims to create a comprehensive study protocol for the development of a clinical practice guideline to research, manage, and share the decision-making in patients with chronic subdural hematoma (cSDH). The protocol outlines a rigorous process involving a steering committee, thematic working groups, patient, carer, and lay panel, as well as collaboration with relevant professional bodies and organizations. While the manuscript outlines the search methods and criteria for evidence selection, it lacks specific details on how datasets will be presented. The authors should consider providing clarity on the format and accessibility of datasets to ensure transparency and facilitate replication by others. This may include specifying the databases used, the search strategy employed, and any tools or software for data analysis. 

**Recommendations for Improvement:**

1. **Enhance Detail on Datasets:** Provide explicit details on the datasets used, including the databases searched, search strategies employed, and any tools or software for data extraction and analysis. This will facilitate transparency and replication.
2. **Specify Data Extraction Template:** Clearly outline the data extraction template used for extracting cohort characteristics. This will assist others in understanding the variables considered and ensure consistency in data extraction.
3. **Clarify Tools for Analysis:** Specify the tools or software used for data analysis, synthesis, and presentation. This information is crucial for researchers aiming to replicate or build upon the study.
4. **Improve Presentation of Datasets:** Clearly describe how datasets will be presented, ensuring usability and accessibility. Consider including details on the format, structure, and any supplementary materials provided.
5. **Detail Implementation of Delphi Exercise **Elaborate on the process of hosting the Delphi exercise on Thiscovery, addressing how participant responses will be managed and the platform's functionality. Provide information on the structure and content of the Delphi questionnaires to ensure clarity and understanding among participants.
6. **Specify External Review Process:** Outline the criteria for selecting external reviewers, ensuring diversity in expertise and perspectives. Clearly define the process of collecting feedback from external reviewers, including the use of Likert scales and free-text comments.

Is the study design appropriate for the research question?

Yes

Is the rationale for, and objectives of, the study clearly described?

Yes

Are sufficient details of the methods provided to allow replication by others?

Yes

Are the datasets clearly presented in a useable and accessible format?

Partly

Reviewer Expertise:

neurosurgery

I confirm that I have read this submission and believe that I have an appropriate level of expertise to confirm that it is of an acceptable scientific standard, however I have significant reservations, as outlined above.

## References

[1] : Middle meningeal artery embolization without surgical evacuation for chronic subdural hematoma: a single-center experience of 209 cases. *Front Neurol* .2023;14: 10.3389/fneur.2023.1222131 1222131 10.3389/fneur.2023.1222131 37719752 PMC10501738

# Reviewer report: Reviewer response for version 1

**DOI:** 10.21956/wellcomeopenres.20492.r66687

**Published:** 2023-09-20

**Authors:** Hiren Patel

**Affiliations:** 1 Department of Neurosurgery, Salford Royal Foundation Trust, Salford, UK

The authors are a diverse group of clinical experts who try to describe how they are going to develop a guideline for the management of patients with a chronic subdural haematoma (csdh). 

 The rationale for the development of this guideline is that csdh is a common problem and that there are no guidelines available. 

 Although not explicitly stated given the detail in the manuscript, I think (or at least hope) that the authors also want to show openly how the guidelines were developed. It is likely that the evidence base for management of this condition is low and given that consensus based guidelines don't respect the link between quality and strength of recommendation a clear and transparent process of guideline development is important. 

 Overall I found the article long and difficult to follow. I wonder if the article could be written mirroring Figure 1 it may be easier to understand ( including the abstract). There were sections that I felt were unnecessary in relation to the project aim. These included the stakeholder section, table 1, target population preference and views and target users sections. 

 The rigour of guideline development should just be guideline development

 I am not sure that the delphi process needs to be explained in the consensus building delphi exercise section and neither is there a need to describe the Thiscovery platform beyond a reference. 

 I am also not sure what the external review section is meant to mean. 

 The applicability section does not add anything to the article. 

 I would hope that by keeping in the sections specific and relevant to the question of how the guideline is going to be developed the authors will have made transparent the process involved in what is likely to be a low evidence based consensus driven clinical guideline.

Is the study design appropriate for the research question?

Partly

Is the rationale for, and objectives of, the study clearly described?

Partly

Are sufficient details of the methods provided to allow replication by others?

Partly

Are the datasets clearly presented in a useable and accessible format?

Not applicable

Reviewer Expertise:

Brain injury.

I confirm that I have read this submission and believe that I have an appropriate level of expertise to confirm that it is of an acceptable scientific standard, however I have significant reservations, as outlined above.
